# Correlation between sarcopenia and esophageal cancer: a narrative review

**DOI:** 10.1186/s12957-024-03304-w

**Published:** 2024-01-24

**Authors:** Shenglan Li, Kaiqiang Xie, Xiaoxiong Xiao, Pingsheng Xu, Mimi Tang, Dai Li

**Affiliations:** 1 Department of Pharmacy, Xiangya Hospital, Central South University, Changsha, 410008 China; 2 National Clinical Research Center for Geriatric Disorders, Xiangya Hospital, Central South University, Changsha, Hunan China; 3 The Hunan Institute of Pharmacy Practice and Clinical Research, Changsha, 410008 China; 4 Institute of Hospital Pharmacy, Central South University, Changsha, 410008 China; 5 Department of Thoracic Surgery, Xiangya Hospital, Central South University, Changsha, Hunan China; 6 Xiangya Lung Cancer Center, Xiangya Hospital, Central South University, Changsha, Hunan China; 7 Phase I Clinical Trial Center, Xiangya Hospital, Central South University, Changsha, 410008 China

**Keywords:** Esophageal cancer, Sarcopenia, Treatment, Prognosis

## Abstract

**Background:**

In recent years, the research on the relationship between sarcopenia before and after the treatment of esophageal cancer, as well as its impact on prognosis of esophageal cancer, has increased rapidly, which has aroused people’s attention to the disease of patients with esophageal cancer complicated with sarcopenia. This review examines the prevalence of sarcopenia in patients with esophageal cancer, as well as the relationship between sarcopenia (before and after surgery or chemotherapy) and prognosis in patients with esophageal cancer. Moreover, we summarized the potential pathogenesis of sarcopenia and pharmacologic and non-pharmacologic therapies.

**Methods:**

A narrative review was performed in PubMed and Web of Science using the keywords (“esophageal cancer” or “esophageal neoplasm” or “neoplasm, esophageal” or “esophagus neoplasm” or “esophagus neoplasms” or “neoplasm, esophagus” or “neoplasms, esophagus” or “neoplasms, esophageal” or “cancer of esophagus” or “cancer of the esophagus” or “esophagus cancer” or “cancer, esophagus” or “cancers, esophagus” or “esophagus cancers” or “esophageal cancer” or “cancer, esophageal” or “cancers, esophageal” or “esophageal cancers”) and (“sarcopenia” or “muscular atrophy” or “aging” or “senescence” or “biological aging” or “aging, biological” or “atrophies, muscular” or “atrophy, muscular” or “muscular atrophies” or “atrophy, muscle” or “atrophies, muscle” or “muscle atrophies”). Studies reporting relationship between sarcopenia and esophageal cancer were analyzed.

**Results:**

The results of the review suggest that the average prevalence of sarcopenia in esophageal cancer was 46.3% ± 19.6% ranging from 14.4 to 81% and sarcopenia can be an important predictor of poor prognosis in patients with esophageal cancer. Patients with esophageal cancer can suffer from sarcopenia due to their nutritional deficiencies, reduced physical activity, chemotherapy, and the effects of certain inflammatory factors and pathways. When classic diagnostic values for sarcopenia such as skeletal muscle index (SMI) are not available clinically, it is also feasible to predict esophageal cancer prognosis using simpler metrics, such as calf circumference (CC), five-count sit-up test (5-CST), and six-minute walk distance (6MWD).

**Conclusions:**

Identifying the potential mechanism of sarcopenia in patients with esophageal cancer and implementing appropriate interventions may hold the key to improving the prognosis of these patients.

**Supplementary Information:**

The online version contains supplementary material available at 10.1186/s12957-024-03304-w.

## Introduction

According to the latest data from the Global Cancer Observatory (GLOBOCAN) database, esophageal cancer (EC) ranks as the eighth most frequently diagnosed cancer and the sixth leading cause of cancer-related death in 2020 [1]. Moreover, the incidence of esophageal cancer has been steadily increasing in recent years. Currently, surgical resection, radiotherapy, and chemotherapy are important means of treating esophageal cancer [2]. For patients with resectable esophageal cancer, according to TNM staging, resectable patients with limited disease of cT1-T2, cN0M0 can be directly treated with surgical resection, whereas patients with locally advanced resectable patients with staging cT3-4 or cN1-3M0 need neoadjuvant chemoradiotherapy or definitive chemoradiotherapy or perioperative chemotherapy before surgery [3]. Esophageal cancer is histologically classified as squamous cell carcinoma (SCC) or adenocarcinoma, with different etiology, pathology, tumor location, treatment, and prognosis [4]. Worldwide, > 85% of all esophageal cancer cases are esophageal squamous cell carcinoma (ESCC) [5]. Alcohol consumption and smoking are responsible for the majority of ESCC cases worldwide, whereas the main risk factors for EAC are gastroesophageal reflux disease (GERD), abdominal obesity, and smoking [4, 5]. ESCC is usually located at or above the tracheal bifurcation, has a tendency for early lymphatic spread, has a poor prognosis, and is the most common histologic type in Eastern Europe, Asia, and Africa, whereas esophageal adenocarcinoma usually involves the lower and middle esophagus and is most common in North America and Western Europe [4, 6]. In addition to this, ESCC is mainly treated with chemoradiotherapy (CRT) with or without surgery. Adenocarcinoma (AC), on the other hand, is usually treated with induction therapy and surgical resection, although the optimal induction regimen is controversial [7]. Unfortunately, since most patients with esophageal cancer are diagnosed at an advanced stage, the overall 5-year survival rate for esophageal cancer remains disappointingly low, with less than 20% of patients surviving beyond this timeframe despite advances in treatment. Therefore, understanding the risk factors associated with esophageal cancer is of paramount importance for public health and clinical decision-making, particularly in terms of risk stratification, screening, and prevention [5].

The concept of muscle function was initially introduced through six consensus definitions in 2010, while the diagnosis of sarcopenia was officially established by the International Classification of Diseases-10 in 2016 [8]. In 2010, the European Working Group on Sarcopenia in Older People (EWGSOP) reported a practical definition of sarcopenia [9]. A similar approach was adopted by the Asian Working Group on Sarcopenia (AWGS) [10]. According to these definitions, sarcopenia is characterized by low muscle mass along with poor muscle function. AWGS demonstrated that sarcopenia should be described as low muscle mass plus low muscle strength and/or low physical performance, and they also recommend outcome indicators for further researches, as well as the conditions that sarcopenia should be assessed. Moreover, they also recommend cutoff values for muscle mass measurements (7.0 kg/m^2^ for men and 5.4 kg/m^2^ for women by using dual X-ray absorptiometry and 7.0 kg/m^2^ for men and 5.7 kg/m^2^ for women by using bioimpedance analysis), handgrip strength (< 26 kg for men and < 18 kg for women), and usual gait speed (< 0.8 m/s). In 2018, EWGSOP revised the consensus and proposed a new definition of sarcopenia, EWGSOP-2 [11]. In this updated definition, EWGSOP-2 includes muscle strength as an important factor and recommends specific cut-off points for the components of sarcopenia. According to EWGSOP-2, sarcopenia is defined by low levels of measures for three parameters: (1) muscle strength, (2) muscle quantity/quality, and (3) physical performance as an indicator of severity [11].

Sarcopenia is characterized as a progressive and generalized skeletal muscle disorder, involving the accelerated loss of muscle mass and function. Notably, sarcopenia has been identified as a prognostic factor for certain cancer types and has been associated with an increased risk of adverse outcomes, including falls, decreased function, frailty, physical disability, and mortality [8, 11]. In the realm of human health, sarcopenia not only heightens the risk of falls and fractures but also impacts activities of daily living, mobility, and has been linked to heart disease, respiratory disease, and cognitive impairment, thereby leading to movement disorders and a diminished quality of life [11]. Recently, sarcopenia has garnered significant attention in the field of oncology and has emerged as a crucial predictor of long-term prognosis in patients with esophageal cancer [12–14]. Given the rising number of elderly patients diagnosed with esophageal cancer, it is worth noting that these individuals often experience cancer-related malnutrition, which contributes to the development of sarcopenia [15]. Additionally, it has been observed that geriatric syndromes such as sarcopenia can impede recovery from esophageal cancer [16].

In this comprehensive review, we delve into various aspects related to sarcopenia in patients with esophageal cancer, including its incidence, prognostic value, the interplay between chemotherapy and sarcopenia, the underlying mechanisms of sarcopenia, therapeutic approaches, and alternative methods for predicting sarcopenia. Our aim is to critically evaluate the combined prognostic impact of factors associated with esophageal cancer and sarcopenia, drawing practical conclusions to support the multidisciplinary management of patients with esophageal cancer and offering fresh insights for the development of therapeutic regimens targeting this disease.

### Prevalence of sarcopenia in esophageal cancer

The prevalence of sarcopenia exhibited considerable variation, depending on the definitions, diagnostic methods, classifications, and cut-off points employed [17]. Notably, several investigations have highlighted disparities in the prevalence of sarcopenia across different regions and age groups [18]. Specifically, sarcopenia affects 5–13% of people aged 60 to 70 and up to 50% of people over 80 [19]. Furthermore, it has been observed that sarcopenia is a prevalent condition within the field of oncology, affecting approximately 35.3% of patients [20]. In a study conducted by Haiducu et al. [21], it was demonstrated that sarcopenia is highly prevalent (43.68%) among individuals with gastrointestinal tumors, with esophageal cancer exhibiting the highest prevalence (70.4%) due to the frequently associated symptom of dysphagia. Additionally, a meta-analysis conducted by Jogiat et al. [13], which encompassed 21 studies and 3966 patients, identified sarcopenia in 1940 individuals, reflecting a prevalence rate of 48.1%. Among the included studies (Table 1), the average prevalence of sarcopenia in esophageal cancer was found to be 46.3% ± 19.6%. However, the prevalence of sarcopenia in patients with esophageal cancer varies considerably due to differences in study populations, age, diagnostic methods, and criteria, and the criteria used to determine the prevalence of sarcopenia varied among the studies in this review, as shown in Table 1, with prevalence rates ranging from 14.4 to 81%. For instance, Tan et al. [22] employed computed tomography (CT) data to retrospectively diagnose sarcopenia in esophageal cancer patients, revealing a sarcopenia prevalence of 75.9%. Conversely, Yoshida et al. [23] conducted a prospective study involving 71 patients with esophageal cancer, utilizing the bioelectrical impedance analysis (BIA) method to diagnose sarcopenia, and reported a sarcopenia prevalence of 40.8% in this cohort. Despite discrepancies in diagnostic criteria and methods, sarcopenia was frequently diagnosed during preoperative examinations in patients with esophageal cancer. Given that esophageal cancer exhibits the highest prevalence of sarcopenia among gastrointestinal tumors, it is imperative to allocate greater attention to this condition in esophageal cancer patients.

**Table 1:** Prediction effect of preoperative sarcopenia on prognosis of esophageal cancer

Author, year	Disease, cases	Age	Sarcopenia (proportion)	Muscle quality measurement	Criteria for sarcopenia	Predictive value	Type of study
Yuichiro Nakashima et al. [24] 2018	EC 341 (total) 166 (age ≥ 65)	NA	170 (49.9%, total) 74 (44.6%, age ≥ 65)	CT	Male: SMI < 47.24^a^, female: SMI < 36.92^a^	In-hospital death↑ (age ≥ 65) Postoperative complications↑ (age ≥ 65)	Retrospective cohort study
Daisuke Makiura et al. [16] 2016	EC 104	NA	29 (27.9%)	BIA	Male: SMI < 7.0^b^, HGS < 26, GS < 0.8 Female: SMI < 5.7^b^, HGS < 18, GS < 0.8	Postoperative pulmonary complications↑	Retrospective cohort study
Jessie A. Elliott et al. [25] 2017	LAEC 192	Mean (SD): 61.6 (9.3)	49 (25.5%)	CT PET-CT	Male: SMI < 52.4^a^, female: SMI < 38.5^a^	Major postoperative complications↑ Postoperative pulmonary complications↑ Prolonged intubation↑ PPCs↑ LOS↑	Prospective cohort study
Satoshi Ida et al. [26] 2015	ESCC 138	NA	61 (44.2%)	BIA	< 90% standard skeletal muscle mass value defined according to the age, sex, and height of each patient	Pulmonary complications↑	Prospective cohort study
Daisuke Makiura et al. [27] 2017	EC 98	Median (IQR): 67 (61–71)	31 (31.6%)	BIA	Male: SMI < 7.0^b^, HGS < 26, GS < 0.8 Female: SMI < 5.7^b^, HGS < 18, GS < 0.8	day unplanned readmission rate↑ OS↓	Prospective cohort study
Pei-yu Wang et al. [28] 2020	EC 212	Mean ± SD: 64.9 ± 7.2	55 (25.9%)	BIA	Male: SMI < 7.0^b^, HGS < 26, GS < 0.8 Female: SMI < 5.7^b^, HGS < 18, GS < 0.8	Overall complications↑ Major complications↑ Delayed hospital discharge↑	Prospective cohort study
Uli Fehrenbach et al. [29] 2021	EC 85	Mean (range): 64.3 (45–83)	58 (68.2%)	CT	Male: L3 SMI ≤ 52.4^a^ Female: L3 SMI ≤ 38.5^a^	LOS↑ Major complications↑	Retrospective cohort study
Makoto Sakai et al. [30] 2020	EC 89	Mean (range): 65 (42–81)	49 (55.1%)	CT	Male: L3 SMI ≤ 52.4^a^ Female: L3 SMI ≤ 38.5^a^	Systemic inflammation↑ OS↓	Retrospective cohort study
Shinya Yoshida et al. [23] 2021	EC 71	Mean (range): 67 (59–72)	29 (40.8%)	BIA	Male: SMI < 7.0^b^ Female: SMI < 5.7^b^	Postoperative complications↑ LOS↑	Prospective cohort study
Jinxin Xu et al. [31] 2019	EC 141	Mean ± SD: 59.7 ± 6.8	73 (51.8%)	CT	Male: L3 SMI ≤ 52.4^a^ Female: L3 SMI ≤ 38.5^a^	Postoperative complications↑	Retrospective cohort study
D. Soma et al. [32] 2018	ESCC 102	Mean: 67.3	45 (44.1%)	CT	Male: SMI < 43^a^, if BMI < 25 SMI < 53^a^, if BMI ≥ 25 Female: SMI < 41^a^	Postoperative respiratory complications↑	Retrospective cohort study
Takuya Fukushima et al. [33] 2023	EC 274	Median (IQR): 65.5 (58.0–71.0)	204 (74.5%)	CT	Male: L3 SMI ≤ 52.4^a^ Female: L3 SMI ≤ 38.5^a^	Postoperative pneumonia↑	Retrospective cohort study
Andrea Cossu et al. [34] 2023	EC 223	Median (range): 62.7 (29–85)	152 (68.1%)	CT	Male: SMI ≤ 52.4^a^ Female: SMI ≤ 38.5^a^	Postoperative 90-day mortality↑	Retrospective cohort study
Bethsabee Benadon et al. [35] 2020	EC 104	Mean ± SD (min–max): 63 ± 12 (20.2–87)	84 (81%) Female: 26 (81.2%) Male: 58 (80.6%)	CT	According to international data: Male: SMI < 43^a^, if BMI < 25 SMI < 53^a^, if BMI ≥ 25 Female: SMI < 41^a^ Mean SMI as cut-off value: Male: SMI < 46^a^ Female: SMI < 35^a^	OS (male)↓	Retrospective cohort study
Kensuke Kudou et al. [36] 2017	EGJC 59	NA	19 (32.2%)	CT	Male: SMI < 43^a^, if BMI < 25 SMI < 53^a^, if BMI ≥ 25 Female: SMI < 41^a^	OS↓ RFS↓	Retrospective cohort study
Xiang Tan et al. [22] 2021	EC 158	NA	120 (75.9%)	CT	Male: L3 SMI ≤ 35.4^a^ Female: L3 SMI ≤ 32.96^a^	OS↓	Retrospective cohort study
Keita Takahashi et al. [37] 2021	EC 229	NA	35 (15.3%)	CT	Male: SMI < 43^a^, if BMI < 25 SMI < 53^a^, if BMI ≥ 25 Female: SMI < 41^a^	OS↓ RFS↓	Retrospective cohort study
Keijiro Sugimura et al. [38] 2022	EC 363	NA	139 (38.2%)	BIA	Male: SMI < 7.0^b^ Female: SMI < 5.7^b^	Poor survival↑	Retrospective cohort study
Takashi Nakayama et al. [39] 2021	EC 63	Mean ± SD: 66.27 ± 7.96	44 (69.84%)	CT	Male: L3 PMI < 6.36 Female: L3 PMI < 3.92	OS↓ DFS↓	Retrospective cohort study
Yohei Ozawa et al. [40] 2019	ESCC 82	Mean ± SD: 63.5 ± 7.5	21 (25.6%)	CT	The sex-specific 25th percentile for the PMI as cut-off value	DFS↓	Retrospective cohort study
Huajian Peng et al. [41] 2021	ESCC 121	NA	65 (53.7%)	CT	Male: L3 SMI ≤ 52.4^a^ Female: L3 SMI ≤ 38.5^a^	OS↓	Retrospective cohort study
Connor J. Wakefield et al. [42] 2021	LAEC 52	Median (IOR): 65 (57–70)	39 (75%)	CT	Male: SMI < 43^a^, if BMI < 25 SMI < 53^a^, if BMI ≥ 25 Female: SMI < 41^a^	OS↓ DFS↓	Retrospective cohort study
Matevz Srpcic et al. [43] 2020	EC 139	Mean ± SD: 63.9 ± 9.5 Range: 30–83	23 (16.5%)	CT	Male: SMI < 43.1 Female: SMI < 32.7^a^	OS↓	Prospective cohort study
J. Oguma, S. Ozawa et al. [44] 2019	SESCC 194	Mean (range): 64.1 (43–86)	28 (14.4%)	CT	Male: L3 SMI ≤ 52.4^a^ Female: L3 SMI ≤ 38.5^a^	OS↓ DFS↓ PPC↑	Retrospective cohort study
Ulf Zeuge et al. [45] 2023	MEC 202	Median (range): 62 (32–88)	103 (51%)	CT	Male: SMI < 43^a^, if BMI < 25 SMI < 53^a^, if BMI ≥ 25 Female: SMI < 41^a^	PFS↓ OS↓	Retrospective cohort study
Miho Yamamoto et al. [46] 2023	ESCC 97	Median (range): 68 (42–81)	44 (46.4%)	CT	SMI < 41^a^	RFS↓ OS↓	Retrospective cohort study
Ricarda Hinzpeter et al. [47] 2023	GEC, EC 128	Mean ± SD: 63.5 ± 11.7 Range: 29–91	60 (47%)	PET-CT CT	Male: SMI < 37.5^a^ Female: SMI < 29.7^a^	PFS↓ OS↓	Retrospective cohort study

*SMI* Skeletal muscle mass index (^a^: cm^2^/m^2^, ^b^: kg/m^2^), *BMI* Body mass index (kg/m^2^), *PMI* Psoas muscle index (kg/m^2^), *IQR* Interquartile range, *HGS* Hand grip strength (kg), *ASM* Appendicular skeletal muscle mass (kg), *LAEC* Locally advanced esophageal cancer, *ESCC* Esophageal squamous cell carcinoma, *OS* Overall survival, *DFS* Disease-free survival, *RFS* Recurrence-free survival, *PFS* Progress-free survival, *GEC* Gastroesophageal cancer, *CT* Computed tomography, *BIA* Bioelectrical impedance analysis, *GS* Gait speed (m/s), *PPC* Postoperative pulmonary complications, *LOS* Length of stay, *EGJC* Esophagogastric junction carcinoma, *UGC* Upper gastric cancer, *MEC* Metastatic esophageal cancer, *NA* No available

### The role of sarcopenia in the prognosis of surgical treatment of esophageal cancer

#### Relationship between preoperative muscle loss and prognosis in esophageal cancer

Sarcopenia, a condition characterized by the loss of muscle mass, has a significant impact on the postoperative prognosis of esophageal cancer. Numerous studies have demonstrated that preoperative sarcopenia not only increases the risk of complications such as pulmonary issues and mortality in older adults, but also leads to extended hospital stays and reduced survival rates. In a retrospective study by Elliott et al. [25], it was discovered that preoperative sarcopenia independently predicted an increase in the Charlson Comorbidity Index (CCI), prolonged length of hospital stay, major postoperative complications, postoperative pulmonary complications (PPC), pneumonia, and prolonged intubation time. Similarly, a retrospective study conducted by Fehrenbach et al. [29] revealed that esophageal cancer patients with comorbid sarcopenia faced a higher risk of major complications and prolonged hospitalization, while obese patients with sarcopenia were significantly more likely to experience pneumonia and extended hospital stays. Nakashima et al. [24] conducted a study on elderly patients with esophageal cancer and found that sarcopenia in this demographic was associated with a higher incidence of anastomotic fistulae and in-hospital death. Another prospective study by Makiura et al. [27] demonstrated that patients with skeletal sarcopenia had a significantly higher rate of unplanned 90-day readmission, with sarcopenia itself being an independent predictor of this outcome according to multivariate logistic regression analysis.

Apart from its impact on surgical complications, preoperative sarcopenia has also been linked to long-term prognosis. Makiura et al. [27] found that sarcopenia reduced overall survival (OS) according to log-rank tests. Another retrospective study [44] identified sarcopenia as an independent prognostic factor affecting both OS and disease-free survival (DFS). Sugimura et al. [38] conducted a study involving 363 patients who underwent esophagectomy and discovered that low preoperative skeletal muscle index (SMI) was associated with poor long-term survival. Additionally, Takahashi et al. [37] observed that preoperative sarcopenia decreased postoperative OS and recurrence-free survival (RFS). Given its implications for postoperative complications and prognosis in esophageal cancer, sarcopenia has emerged as a crucial prognostic factor. The studies listed in Table 1 provide evidence of sarcopenia’s association with complications and prognosis in esophageal cancer. Consequently, the routine evaluation and accurate diagnosis of sarcopenia in esophageal cancer patients can assist clinicians in tailoring treatment plans, providing timely nutritional support, and ultimately improving short-term and long-term patient outcomes, as well as the overall prognosis of esophageal cancer.

#### Relationship between postoperative muscle loss and prognosis in esophageal cancer carcinoma

Previous investigations have primarily focused on examining the consequences of preoperative sarcopenia on postoperative complications and prognosis. However, the impact of diminished skeletal muscle mass following esophagectomy in individuals with esophageal cancer on long-term postoperative prognosis remains insufficiently explored [48]. The loss of skeletal muscle mass in the acute phase after surgery may serve as a novel prognostic indicator for long-term outcomes, particularly in highly invasive procedures like ESCC surgery. Notably, it was observed that the 3-year overall survival rate was notably lower in the group with severely reduced total psoas mass index (TPI) compared to the group with mildly reduced TPI [49]. A study by Takahashi et al. [50] found that substantial skeletal muscle loss in esophageal cancer patients after 3 months postoperatively was associated with poorer OS and RFS. Additionally, another study revealed a significant correlation between reduced SMI and a worsened prognosis following esophageal cancer resection [51]. Kudou et al.’s study [52] demonstrated that the development of postoperative sarcopenia in patients with adenocarcinoma of the esophagogastric junction (AEG) and upper gastric cancer (UGC) independently predicted poor overall survival in multivariate analysis. Furthermore, the progression of sarcopenia was found to be indicative of unfavorable recurrence-free survival in patients with AEG and UGC. Another retrospective study [53] indicated that a greater decline in PMI after neoadjuvant chemoradiation therapy (NACRT) and esophagectomy constituted a significant risk factor for overall survival and recurrence-free survival. The prognosis of postoperative muscle loss in esophageal cancer has received limited attention and the timing of postoperative detection of sarcopenia varies considerably across studies, but each of these studies consistently demonstrates a substantial association between postoperative muscle loss or reduced skeletal muscle mass and poor prognosis, and more prospective cohort studies are needed to demonstrate this association.

### Chemotherapy and sarcopenia

#### Chemotherapy-induced sarcopenia

Multimodal neoadjuvant concurrent chemoradiotherapy (CCRT) has gained traction in the treatment of esophageal cancer, specifically in cases of ESCC and esophageal adenocarcinoma [54]. The incidence of sarcopenia can rise by 17% from neoadjuvant chemotherapy until the completion of treatment in individuals with tumors [55]. A particular study [56] underscored the negative impact of chemotherapy-related adverse events, such as fatigue, loss of appetite, nausea, vomiting, and diarrhea, on food intake, physical activity, and ultimately, the severe loss of muscle mass. In a retrospective cohort study conducted by Halliday et al. [57], significant reductions in body weight, BMI, skeletal muscle (SM) area, skeletal muscle index (SMI), visceral adipose tissue (VAT), and total adipose tissue (TAT) were observed following neoadjuvant therapy. Fujihata et al. [58] investigated esophageal cancer-related skeletal muscle wasting (SMW) during neoadjuvant chemotherapy (NAC) and discovered a declining trend in SMI and body weight among patients with esophageal cancer during NAC treatment. Furthermore, decreasing SMI was found to be associated with a higher incidence of postoperative anastomotic fistula. In another retrospective study [59], it was determined that the mean change in total psoas area (TPA) of patients before and after neoadjuvant chemotherapy was 7.3% (6.8%). During neoadjuvant therapy, 43 (81.1%) patients experienced some degree of psoas loss. The target population in certain studies extends beyond patients with esophageal cancer. For instance, Oflazoglu et al. [60] included a substantial number of patients with primary tumors and assessed various indicators of sarcopenia before chemotherapy, as well as at the third and sixth months following chemotherapy. Their findings indicated a continuous rise in the prevalence of sarcopenia during chemotherapy. The study by Jogiat et al. [61] also corroborated this point. They observed that the prevalence of sarcopenia in patients with esophageal cancer increased from 17.0% before chemotherapy to 38.1% after chemotherapy, nearly doubling the incidence of sarcopenia in this specific patient group.

#### Sarcopenia leads to increased chemotherapy-related toxicity

Due to the narrow therapeutic range of chemotherapeutic agents used for esophageal cancer, it becomes crucial to identify factors that can predict individual variances in chemotherapy toxicity and effectiveness [62]. A retrospective study [63] discovered a significantly higher occurrence of grade 3–4 toxicity among the 184 ESCC patients included, who also had sarcopenia. The major treatment-related toxicities observed in grades 3–4 were leukopenia, neutropenia, esophagitis, and anorexia. Several other studies [64, 65] have yielded similar findings. Furthermore, additional studies [66, 67] have demonstrated that sarcopenia increases the likelihood of dose-limiting toxicity (DLT) and serves as a significant predictor of DLT. Tan et al. [66] conducted a retrospective study specifically focusing on the impact of sarcopenia on dose-limiting toxicity of neoadjuvant chemotherapy in patients with esophagogastric cancer. The study revealed a significant correlation between sarcopenia and DLT, highlighting the necessity of employing various methods to assess skeletal muscle mass in order to predict toxicity and customize chemotherapy dosage. Another retrospective study conducted by Ota et al. [62] also identified sarcopenia as an independent predictor of poor pathological response.

Table 2 lists the studies related to the effect of sarcopenia on chemotherapy in esophageal cancer. Based on the aforementioned studies, it is evident that sarcopenia independently indicates reduced overall survival [14, 63, 68–70], disease-free survival [40, 71], and recurrence-free survival [14] in patients with esophageal cancer who undergo chemotherapy. Furthermore, sarcopenia increases the incidence of toxic reactions [66, 67], mucositis, fever [71], and lymphopenia [70], consequently leading to perioperative complications [68, 72], an elevated risk of postoperative recurrence rates [40], and postoperative mortality [73]. Early implementation of appropriate nutritional intervention prior to treatment may improve prognosis [74].

**Table 2:** Predictive effect of pre-chemotherapy sarcopenia on chemotherapy toxicity

Author, year	Disease, cases	Sarcopenia (proportion)	Chemotherapy regimens	Impact on chemotherapy toxicity or outcomes
Cédric M. Panje et al. [64] 2019	EC 61	18 (29.5%)	DP (docetaxel + cisplatin) with or without cetuximab	Grade ≥ 3 toxicities↑
Ying-Ying Xu et al. [63] 2021	ESCC 184	94 (51.1%)	DP (docetaxel + cisplatin)	Grade 3–4 toxicities (leukopenia, neutropenia, anorexia)↑ OS↓
Chunhou Huang et al. [71] 2020	ESCC 107	65 (67%)	Cisplatin + fluorouracil	Grade 3 to 4 mucositis↑ Grade 3 to 4 fever and neutropenia fever↑ OS↓ DFS↓
Christine Koch et al. [68] 2019	GC, GEJC 83	30 (36.1%)	FLOT (5-flurouracil, leucovorin, oxaliplatin, and docetaxel) EOX (epirubicin, oxaliplatin, and capecitabine) ECX (epirubicin, cisplatin, capecitabine)	Severe perioperative complications↑ Survival↓ Terminated the chemotherapy significantly earlier
B.H.L. Tan et al. [66] 2015	OGC 89	44 (49.4%)	ECX (epirubicin, cisplatin, and capecitabine) CF (cisplatin and 5-FU)	DLT↑
Yohei Ozawa et al. [40] 2019	ESCC 82	21 (25.6%)	Cisplatin + 5-FU	DFS↓ Postoperative recurrence↑
Takayuki Ota et al. [62] 2019	EC 31	16 (51.6%)	FP (cisplatin and 5-fluorouracil (5-FU)) DCF (cisplatin, 5-FU, and docetaxel)	Poor pathological response
Gilbert Z. Murimwa et al. [65] 2017	EC 56	23 (41%)	Cisplatin + fluorouracil	Acute Grade ≥ 3 toxicity (dysphagia, neutropenia, hospitalization during treatment)↑
Poorna Anandavadivelan et al. [67] 2016	EC 72	31 (43%)	Cisplatin/oxaliplatin/carboplatin + 5-fluorouracil	DLT↑
Kostan W. Reisinger et al. [75] 2015	EC 108	60 (56%)	CF (cisplatin and 5-FU) PC (paclitaxel/carboplatin) ECC (epirubicin/cisplatin/capecitabine)	Postoperative mortality↑
Katsuhiko Nara et al. [76] 2023	EC 39	20 (51.3%)	DCF (docetaxel + cisplatin + 5‑fluorouracil)	FN↑
Tsuyoshi Harada et al. [77] 2023	LAEC 188	68 (36.2%)	FP (cisplatin and 5-fluorouracil (5-FU)) DCF (cisplatin, 5-FU, and docetaxel)	RDI↓
Nishi S et al. [72] 2023	EC 36	11 (30.6%)	NA	PP↑
Dónal Michael McSweeney et al. [70] 2023	EC 135	68 (50.4%)	PC (paclitaxel/carboplatin) Cisplatin + capecitabine or carboplatin + capecitabine	OS↓ Radiation-induced lymphopenia↑

*EC* Esophageal cancer, *GC* Gastric cancer, *GEJC* Gastro-esophageal junction cancer, *OS* Overall survival, *DFS* Disease-free survival, *RDI* Relative dose intensity, *LAEC* Locally advanced esophageal cancer, *DLT* Dose limiting toxicity, *OGC* Oesophago-gastric cancer, *FN* Febrile neutropenia, *PP* Postoperative pneumonia, *NA* No available

### Potential mechanisms of esophageal cancer-associated sarcopenia

In individuals afflicted with EC, sarcopenia may arise as a result of nutritional deficiencies caused by dysphagia, pain, systemic inflammation, and an augmented metabolic rate [63].

#### Malnutrition

Malnutrition is a condition characterized by changes in body composition and cellular mass resulting from inadequate nutrient intake or absorption. This deficiency leads to compromised physical and mental abilities, with involuntary weight loss being one of its prominent manifestations [78]. Moreover, malnutrition has been linked to the mechanisms of sarcopenia [79]. The development of malnutrition-induced sarcopenia is influenced by various factors, including abnormal protein and energy metabolism in tumor cells, inflammation, impaired immunity, and cancer-related symptoms such as fatigue, pain, cough, and loss of appetite [14]. Gastrointestinal cancers, such as esophageal and gastric cancer, often exhibit reduced gastrointestinal function, particularly affecting swallowing and digestion in esophageal cancer. Consequently, protein intake is adversely affected. Among all types of cancer, esophageal cancer has one of the highest prevalence rates of increased nutritional risk, exceeding 60% [79].

#### Lack of exercise lifestyle

Lack of physical activity is widely acknowledged as the primary risk factor for sarcopenia [19]. The decline in skeletal muscle mass and strength becomes apparent around the age of 40, and the incidence of sarcopenia increases with age. Furthermore, the loss of muscle mass and strength accelerates as individuals grow older [80]. Sedentary individuals experience a more significant decline in muscle fiber and strength compared to those who are physically active [19]. There is often a decrease in physical activity after esophagectomy [81]. Therefore, patients with esophageal cancer should also pay attention to sarcopenia due to reduced physical activity.

#### Inflammation

Inflammation is a response triggered by tissue dysfunction or disturbances in the body’s balance, and it is believed to underlie various physiological and pathological processes [82]. Inflammatory cells and mediators are present in the microenvironment of most tumors [83]. Pro-inflammatory cytokines, including interleukin-1 (IL-1), IL-6, and tumor necrosis factor-α (TNF-α), have been identified as mediators of anorexia and the breakdown of skeletal muscle protein, which are crucial components of cancer malignant stroma [12]. These cytokines contribute to muscle deterioration by promoting the infiltration of inflammatory cells through NF-κB [84], esophageal cancer has been shown to activate NF-κB [73], and this activation of the NF-κB pathway is paralleled by a simultaneous increase in IL-1, IL-6, and TNF-α [85]. TNF-α exacerbates catabolism (protein loss, insulin resistance), impairs muscle contraction, disrupts myogenesis, and ultimately leads to muscle weakness [86]. The chronic inflammatory response not only diminishes skeletal muscle function but also triggers a vicious cycle by inducing skeletal muscle tissue dysfunction, thus accelerating the progression of sarcopenia [82].

#### Chemotherapy causes sarcopenia

A multicenter study demonstrated that neoadjuvant chemoradiation in esophageal cancer patients increased the percentage of sarcopenia [64], and another study found that 32.5% of esophageal cancer patients had body composition changes during NAC (patients with ≥ 3% increase in visceral fat mass (VFM) and ≥ 3% decrease in PMI) [87]. In addition, a systematic review noted that esophageal cancer patients receiving chemotherapy are at risk for severe loss of skeletal muscle mass [88]. Several adverse effects of chemotherapy, such as fatigue, loss of appetite, nausea, vomiting, diarrhea, taste disturbances, anorexia, mucositis, and dysphagia, can negatively impact food intake, physical activity, and ultimately result in significant muscle mass loss [89]. Insulin-like growth factor-1 (IGF-1) is a well-studied activator of muscle hypertrophy, and its receptor, IGF-1 R, mediates protein synthesis activation [90]. The IGF 1-PI3K-Akt/PKB-mTOR pathway positively regulates muscle growth [91]. However, certain chemotherapeutic agents, such as cisplatin, one of the commonly used drugs in chemotherapy for esophageal cancer, can reduce IGF-1 protein levels by approximately 85% and inhibit IGF-1/PI3K/Akt signaling in skeletal muscle [92]. Consequently, the downregulation of IGF-1 expression in skeletal muscle during chemotherapy may be a significant factor contributing to the development of muscle weakness in cancer patients [92]. Chemotherapeutic drugs such as cisplatin and irinotecan directly induce muscle loss by activating the transcription factor NF-κB, which upregulates ubiquitin and the proteasome, leading to increased protein breakdown and the release of inflammatory cytokines (IL-1 β, IL-6, and TNF-α). These inflammatory cytokines further enhance the activity of E3 ligase (atrogin-1) and promote ubiquitin-mediated protein degradation [86].

#### Other signaling pathways

mTOR is a crucial regulator of skeletal muscle mass [90], and the IGF1-PI3K-Akt/PKB-mTOR pathway positively regulates muscle growth [91]. mTOR also plays a significant role in mitochondrial metabolism, protein synthesis enhancement, and the promotion of mitochondrial biosynthesis and adipogenesis. The tumor suppressors liver kinase B1 (LKB1) and AMPK regulate cell growth in response to changes in environmental nutrient levels and generally downregulate the mTOR pathway, resulting in reduced protein synthesis and the development of sarcopenia [93].

### Predicting esophageal cancer prognosis with a simple indicator in the diagnosis of sarcopenia

In addition to employing SMI values to define sarcopenia and determine esophageal cancer prognosis in most studies, numerous researchers have utilized alternative methods as prognostic indicators for esophageal cancer. For instance, several studies have showcased the predictive role of skeletal muscle mass loss in determining esophageal cancer prognosis [51, 87, 88, 94]. Moreover, measurements of the total psoas major area (TPA) [59] and the psoas muscle index [53] have been utilized as surrogate markers of sarcopenia to predict postoperative complications, overall survival (OS), and recurrence-free survival (RFS) in esophageal cancer patients. Furthermore, Kurita conducted several studies [95–97] employing hand grip strength (HGS) and the five-count sit-up test as predictors of esophageal cancer prognosis, revealing that HGS and 5-CST can significantly predict complications such as postoperative pneumonia. A retrospective study by Zhou et al. [98] also identified low subcutaneous fat as a risk factor for increased mortality. Additionally, the sarcopenia index, specifically the serum creatinine/cystatin C ratio, has been employed to predict prognosis in esophageal cancer patients and has been associated with postoperative complications and long-term survival [99]. This index has also demonstrated similar associations in other types of cancer [100–103]. Alongside grip strength and 5-CST, gait speed (GS) and six-minute walk distance, which are diagnostic criteria for sarcopenia, can be utilized to determine a patient’s prognosis. A prospective study analyzing 922 elderly men revealed that slow gait speed increased the risk of death in elderly male cancer patients [104]. Multiple other studies have demonstrated that GS and 6MWD can predict survival [105, 106] and complications [107, 108]. Additionally, some studies have proposed the use of calf circumference (CC) as a diagnostic indicator for sarcopenia to enhance diagnostic accuracy [109]. A prospective study by Sousa et al., which included 250 patients, discovered that a low CC predicted the risk of death in cancer patients [110]. Several other studies have also indicated that CC can serve as a simpler, faster, and cost-effective measurement to rapidly screen patients at risk of death [111–113].

Besides assessing the presence of sarcopenia according to diagnostic criteria and gauging its prognostic significance, researchers have been particularly intrigued by the extent of skeletal muscle mass reduction during treatment or post-surgery. In instances where standardized tests fail to meet the criteria for diagnosing sarcopenia, employing alternative, efficient methods like HGS, 5-SCT, GS, 6MWD, and CC to predict prognosis is highly desirable (Table 3).

**Table 3:** A simpler way replaces sarcopenia to predict esophageal cancer prognosis

Author, year	Disease, cases	Measurement indicators	Specific measurement methods	Predictive value
James M. Halle-Smith et al. [114] 2022	EC 26	A reduced preoperative BMI	BMI = weight/height^2^	CL↑
Jianjian Qiu et al. [115] 2023	ESCC 160	BMI	The cut-off value for BMI was 19.51	OS↓ PFS↓
Liu Jiang et al. [116] 2023	ESCC 158	BMI	The cut-off value for BMI was 18.5	OS↓ PFS↓
Alexandra N Townsend et al. [117] 2023	EC 2544	BMI	The cut-off value for BMI was 18.5	Postoperative complications↑
Daisuke Kurita et al. [95] 2020	EC 161	Low HGS	Low HGS: < 27 for men and < 16 for women	Postoperative pneumonia↑
Daisuke Kurita et al. [96] 2022	EC 201	Low HGS	Low HGS: < 27 for men and < 16 for women	Early postoperative aspiration↑ Impairment of airway protective reflexes↑
Chao Zheng et al. [99] 2022	EC 203	SI	Sarcopenia index (SI = creatinine/cystatin C × 100)	Postoperative complications↑ Long-term survival↓
Sugimura Keijiro et al. [118] 2022	EC 363	6MWD	The cut-off value for 6MWD was 400	The rate of death↑
Kondo Shin et al. [106] 2021	EC 108	6MWD	The cut-off value for 6MWD was 480	OS↓ RFS↓
Takayuki Inoue et al. [108] 2020	EC 111	6MWD	The cut-off value for 6MWD was 454	Postoperative complications↑
Daisuke Kurita et al. [97] 2022	EC 222	5-CST	The 5-CST measures the amount of time needed for a patient to rise five times from a seated position with their arms folded across the chest as quickly as possible	Postoperative pneumonia↑

*CL* Chyle leak, *ESCC* Esophageal squamous cell carcinoma, *OS* Overall survival, *RFS* Recurrence-free survival, *PFS* Progress-free survival, *BMI* Body mass index (kg/m^2^), *HGS* Hand grip strength (kg), *SI* Sarcopenia index, *5-CST* Five-time chair stand test, *6MWD* 6-min walk distance (m)

### Treatment of sarcopenia

#### Non-pharmacological treatment

A comprehensive assessment has concluded that the implementation of suitable physical activity, strength training, and nutritional interventions, coupled with a stable biological clock, holds the potential to enhance skeletal muscle growth, decelerate skeletal muscle deterioration, and ameliorate symptoms associated with sarcopenia [119].

The absence of physical activity has been linked to a decline in muscle strength and mass. Hence, exercise programs are considered the fundamental element in the treatment of sarcopenia, as they can mitigate muscle loss by reducing the activation of NF-κB [120]. Short-term resistance exercise has demonstrated the ability to enhance the synthesis of proteins in skeletal muscle, bolstering its ability and capacity [19]. Long-term resistance training, on the other hand, has proven to enhance both muscle strength and mass [121]. A systematic review has revealed that resistance training, as well as a combination of resistance training with other exercises, can enhance muscle strength and gait speed (GS) [122]. Furthermore, specific strength exercises contribute to the amelioration of muscle function and neuromuscular adaptations [119]. In a cohort study conducted by Ziegler et al. [121], subjects were randomly assigned to either a 1-year heavy resistance training (HRT) group or a control group (CON). After 12 months of training, the HRT group exhibited significantly higher isometric and dynamic muscle strength compared to the control group.

Certain nutritional interventions, such as the consumption of high-protein or essential amino acids like leucine, in conjunction with resistance training, have the potential to delay skeletal muscle loss observed in sarcopenia [119]. Specific dietary patterns, including the consumption of adequate protein such as leucine-containing protein supplements or whey protein, vitamin D, antioxidant nutrients, and long-chain polyunsaturated fatty acids, have proven beneficial in the prevention and improvement of sarcopenia [18]. Esophageal cancer patients undergoing esophagectomy require careful postoperative nutritional monitoring due to fasting requirements. Studies have indicated that more than half of the patients exhibited inadequate oral intake upon discharge [123]. Therefore, for patients with esophageal cancer, enteral nutrition is a preferred option to meet internal nutritional requirements, while parenteral nutrition can be considered if enteral nutrition is insufficient or undesirable [124]. It has been observed that patients receiving nutritional management exhibited higher serum total protein and albumin levels, fewer postoperative adverse events, and lower hospitalization costs compared to those following a conventional diet [125]. Additionally, declines in weight, BMI, and appendicular skeletal muscle mass index (ASMI) were significantly reduced, leading to improvements in patients’ quality of life and fatigue status [74]. Another retrospective study [126] noted that adults who received early nutritional support during neoadjuvant therapy experienced less weight loss at 12 months after esophagectomy compared to those who received oral nutritional support after surgery. Therefore, providing appropriate nutritional support at the correct time is of utmost importance for patients with esophageal cancer.

Skeletal muscle, as a peripheral organ, is regulated by the biological clock [127], a mechanism that fosters skeletal muscle growth and maintains homeostasis within the body [119]. A review encompassing numerous studies on genetic strategies involving targeted gene failure in mice specifically related to skeletal muscle has revealed that many circadian mutants exhibit muscle defects [127]. A deeper understanding of the molecular clock of skeletal muscle and its relationship with muscle-skeletal interactions could yield valuable insights into sarcopenia. Consequently, more effective intervention strategies (e.g., exercise and dietary restrictions) can be developed based on the biological clock [128]. This, in turn, could help prevent muscle loss during aging or in chronic diseases that may lead to sarcopenia by preserving and promoting the proper functioning of intrinsic muscle clock mechanisms [129].

#### Pharmacologic treatment

Currently, there are no FDA-approved treatments available for sarcopenia [19]. However, a comprehensive review has revealed the existence of several recommended agents with varying degrees of effectiveness. These include growth hormone, anabolic or androgenic steroids, selective androgen receptor modulators, protein anabolic agents, appetite stimulants, myostatin inhibitors, activating II receptor drugs, β-receptor blockers, angiotensin-converting enzyme inhibitors, and troponin activators [18].

In a study conducted by Hirani et al. [130], it was discovered that low levels of vitamin D were significantly associated with the occurrence of sarcopenia. Therefore, maintaining adequate vitamin D levels may reduce the incidence of this condition. Another cross-sectional study found a correlation between growth hormone (GH) and insulin-like growth factor (IGF-1) with sarcopenia in older adults. This suggests that the use of IGF-1 and GH may potentially increase skeletal muscle mass [131]. Additionally, it was observed that individuals taking metformin [132] or statins [133] had a lower risk of developing sarcopenia compared to those not taking these medications. This demonstrates the potential protective effect of metformin against sarcopenia. Furthermore, it appears that statins may also prevent the development of sarcopenia, with higher doses showing a more pronounced preventive effect. A prospective study involving 740 older adults revealed a significant positive correlation between calcium intake and appendicular lean mass (ALM) [134]. Animal studies have also suggested that losartan may slow down muscle degeneration, promote clinical benefits, and provide protection for patients with sarcopenia [135, 136].

Despite the existence of studies showcasing the positive effects of the aforementioned drugs in sarcopenia patients, their efficacy remains a subject of controversy. Furthermore, the optimal dosage and potential side effects of these drugs require further investigation through additional studies. The pharmacological treatment of sarcopenia necessitates more extensive exploration and clinical trials to scientifically evaluate the efficacy of these drugs.

## Conclusions and future perspectives

To date, the majority of studies investigating sarcopenia in esophageal cancer patients have primarily relied on retrospective approaches, severely constraining their ability to comprehensively depict patient populations. Consequently, our understanding of the underlying mechanisms linked to heightened adverse outcomes remains limited [137]. Hence, it is imperative to conduct more prospective evaluations on sarcopenia in individuals afflicted with esophageal cancer. These evaluations will enable us to establish a more profound comprehension of the correlation between sarcopenia, characterized by the depletion of skeletal muscle mass or strength, and adverse outcomes or post-treatment complications. Furthermore, they will facilitate the development of precise and personalized interventions based on the findings, thereby enhancing outcomes in high-risk populations [137]. By performing requisite assessments of sarcopenia in esophageal cancer patients, we can devise optimal treatment strategies that rectify the sarcopenic condition prior to surgery or chemotherapy through nutritional support and exercise, adjuvant therapy, and meticulous postoperative monitoring [138]. This comprehensive approach aims to augment the quality of life for patients with esophageal cancer while simultaneously alleviating the healthcare burden on society.

### Supplementary Information

**Additional file 1.** This supplement describes the various parts of the manuscript in detail according to the major sections and topics required by the PRISMA guidelines.

## Data Availability

Not applicable.

## Abbreviations

CC: Calf circumference
5-CST: Five-count sit-up test
6MWD: Six-minute walk distance
GLOBOCAN: Global cancer observatory
EC: Esophageal carcinoma
SCC: Squamous cell carcinoma
ESCC: Esophageal squamous cell carcinoma
GERD: Gastroesophageal reflux disease
CRT: Chemoradiotherapy
AC: Adenocarcinoma
EWGSOP: European working group on sarcopenia in older people
AWGS: Asian working group on sarcopenia
CT: Computed tomography
BIA: Bioelectrical impedance analysis
CCI: Charlson comorbidity index
PPC: Postoperative pulmonary complications
OS: Overall survival
DFS: Disease-free survival
SMI: Skeletal muscle mass index
RFS: Recurrence-free survival
BMI: Body mass index
PMI: Psoas muscle index
IQR: Interquartile range
HGS: Hand grip strength
ASM: Appendicular skeletal muscle mass
LAEC: Locally advanced esophageal cancer
PFS: Progress-free survival
GEC: Gastroesophageal cancer
GS: Gait speed
LOS: Length of stay
EGJC: Esophagogastric junction carcinoma
UGC: Upper gastric cancer
MEC: Metastatic esophageal cancer
NA: No available
TPI: Total psoas mass index
AEG: Adenocarcinoma of esophagogastric junction
UGC: Upper gastric cancer
NACRT: Neoadjuvant chemoradiation therapy
CCRT: Concurrent chemoradiotherapy
SM: Skeletal muscle
VAT: Visceral adipose tissue
TAT: Total adipose tissue
SMW: Skeletal muscle wasting
NAC: Neoadjuvant chemotherapy
TPA: Total psoas area
DLT: Dose-limiting toxicity
GC: Gastric cancer
GEJC: Gastro-esophageal junction cancer
RDI: Relative dose intensity
OGC: Oesophago-gastric cancer
FN: Febrile neutropenia
PP: Postoperative pneumonia
IL-1: Interleukin-1
TNF-α: Tumor necrosis factor-α
VFM: Visceral fat mass
IGF-1: Insulin-like growth factor-1
LKB1: Liver kinase B1
CL: Chyle leak
SI: Sarcopenia index
HRT: Heavy resistance training
CON: Control group
ASMI: Appendicular skeletal muscle mass index
GH: Growth hormone
ALM: Appendicular lean mass
