# Supplementary material for: Correlation between sarcopenia and esophageal cancer: a narrative review
Source: World J Surg Oncol. 2024 Jan 24;22:27. doi: 10.1186/s12957-024-03304-w (PMC10809562; doi:10.1186/s12957-024-03304-w)
Supplement: Supplementary file 1 — Additional file 1. This supplement describes the various parts of the manuscript in detail according to the major sections and topics required by the PRISMA guidelines. [file 12957_2024_3304_MOESM1_ESM.pdf]

| Section and Topic | Item | Checklist item | Section of manuscript | Location where item is reported |
|---|---|---|---|---|
| **Title** | 1 | Identify the report as a systematic review. | Title | Page1, Correlation between sarcopenia and esophageal cancer: a narrative review |
| **Abstract** | 2 | | | |
| Background | | | | |
| Objectives | | Provide an explicit statement of the main objective(s) or question(s) the review addresses. | Abstract | Page1, " This review examines the prevalence of sarcopenia in patients with esophageal cancer, as well as the relationship between sarcopenia (before and after surgery or chemotherapy) and prognosis in patients with esophageal cancer. Moreover, we summarized the potential pathogenesis of sarcopenia and pharmacologic and non-pharmacologic therapies. " |
| Methods | | | | |
| Eligibility criteria | | Specify the inclusion and exclusion criteria for the review. | Abstract | Page1, " Methods: A narrative review was performed in PubMed and Web of Science using the keywords (“esophageal cancer” or “esophageal neoplasm” or “neoplasm, esophageal” or “esophagus neoplasm” or “esophagus neoplasms” or “neoplasm, esophagus” or “neoplasms, esophagus” or “neoplasms, esophageal” or “cancer of esophagus” or “cancer of the esophagus” or “esophagus cancer” or “cancer, esophagus” or “cancers, esophagus” or |

| | | | | |
|---|---|---|---|---|
| | | | | "esophagus cancers" or "esophageal cancer" or "cancer, esophageal" or "cancers, esophageal" or "esophageal cancers") and ("sarcopenia" or "muscular atrophy" or "aging" or "senescence" or "biological aging" or "aging, biological" or "atrophies, muscular" or "atrophy, muscular" or "muscular atrophies" or "atrophy, muscle" or "atrophies, muscle" or "muscle atrophies"). Studies reporting relationship between sarcopenia and esophageal cancer were analyzed. " |
| Information sources | | Specify the information sources (e.g. databases, registers) used to identify studies and the date when each was last searched. | Abstract | Page1, " Methods: A narrative review was performed in PubMed and Web of Science using the keywords ("esophageal cancer" or "esophageal neoplasm" or "neoplasm, esophageal" or "esophagus neoplasm" or "esophagus neoplasms" or "neoplasm, esophagus" or "neoplasms, esophagus" or "neoplasms, esophageal" or "cancer of esophagus" or "cancer of the esophagus" or "esophagus cancer" or "cancer, esophagus" or "cancers, esophagus" or "esophagus cancers" or "esophageal cancer" or "cancer, esophageal" or "cancers, esophageal" or "esophageal |

| | | | | |
|---|---|---|---|---|
| | | | | cancers”) and (“sarcopenia” or “muscular atrophy” or “aging” or “senescence” or “biological aging” or “aging, biological” or “atrophies, muscular” or “atrophy, muscular” or “muscular atrophies” or “atrophy, muscle” or “atrophies, muscle” or “muscle atrophies”). Studies reporting relationship between sarcopenia and esophageal cancer were analyzed. " |
| Risk of bias | | Specify the methods used to assess risk of bias in the included studies. | | not applicable |
| Synthesis of results | | Specify the methods used to present and synthesise results. | | not applicable |
| Results | | | | |
| Included studies | | Give the total number of included studies and participants and summarise relevant characteristics of studies | | not applicable |
| Synthesis of results | | Present results for main outcomes, preferably indicating the number of included studies and participants for each. If meta-analysis was done, | Abstract | Page2, "Results: The results of the review suggest that the average prevalence of sarcopenia in esophageal cancer was 46.3 % ± 19.6% ranging from 14.4% to 81% and sarcopenia can be an important predictor of poor prognosis in patients with esophageal cancer. |

| | | | | |
|---|---|---|---|---|
| | | report the summary estimate and confidence/credible interval. If comparing groups, indicate the direction of the effect (i.e. which group is favoured). | | Patients with esophageal cancer can suffer from sarcopenia due to their nutritional deficiencies, reduced physical activity, chemotherapy and the effects of certain inflammatory factors and pathways. When classic diagnostic values for sarcopenia such as skeletal muscle index (SMI) are not available clinically, it is also feasible to predict esophageal cancer prognosis using simpler metrics, such as calf circumference (CC), five-count sit-up test (5-CST), six-minute walk distance (6MWD), etc. " |
| Discussion | | | | |
| Limitations of evidence | | Provide a brief summary of the limitations of the evidence included in the review (e.g. study risk of bias, inconsistency and imprecision). | | not applicable |
| Interpretation | | Provide a general interpretation of the results and important implications. | Abstract | Page2, " Conclusions: Identifying the potential mechanism of sarcopenia in patients with esophageal cancer and implementing appropriate interventions, may hold the key to improving the prognosis of these patients. " |
| **Introduction** | | | | |
| Rationale | 3 | Describe the | Introduction | Page2, the introduction section |

| | | | | |
|---|---|---|---|---|
| | | rationale for the review in the context of existing knowledge. | | introduces the relevant background about esophageal cancer and sarcopenia and the purpose of this review |
| Objectives | 4 | Provide an explicit statement of the objective(s) or question(s) the review addresses. | Introduction | Page4, "In this comprehensive review, we delve into various aspects related to sarcopenia in patients with esophageal cancer, including its incidence, prognostic value, the interplay between chemotherapy and sarcopenia, the underlying mechanisms of sarcopenia, therapeutic approaches, and alternative methods for predicting sarcopenia. Our aim is to critically evaluate the combined prognostic impact of factors associated with esophageal cancer and sarcopenia, drawing practical conclusions to support the multidisciplinary management of patients with esophageal cancer and offering fresh insights for the development of therapeutic regimens targeting this disease. " |
| **Methods** | | | | |
| Eligibility criteria | 5 | Specify the inclusion and exclusion criteria for the review and how studies were grouped for the syntheses. | Abstract | Page1, "Methods: A narrative review was performed in PubMed and Web of Science using the keywords (“esophageal cancer” or “esophageal neoplasm” or “neoplasm, esophageal” or “esophagus neoplasm” or “esophagus neoplasms” or “neoplasm, esophagus” or |

| | | | | |
|---|---|---|---|---|
| | | | | “neoplasms, esophagus” or “neoplasms, esophageal” or “cancer of esophagus” or “cancer of the esophagus” or “esophagus cancer” or “cancer, esophagus” or “cancers, esophagus” or “esophagus cancers” or “esophageal cancer” or “cancer, esophageal” or “cancers, esophageal” or “esophageal cancers”) and (“sarcopenia” or “muscular atrophy” or “aging” or “senescence” or “biological aging” or “aging, biological” or “atrophies, muscular” or “atrophy, muscular” or “muscular atrophies” or “atrophy, muscle” or “atrophies, muscle” or “muscle atrophies”). Studies reporting relationship between sarcopenia and esophageal cancer were analyzed. " |
| Information sources | 6 | Specify all databases, registers, websites, organisations, reference lists and other sources searched or consulted to identify studies. Specify the date when each source was last searched or consulted. | Abstract | Page1, "Methods: A narrative review was performed in PubMed and Web of Science using the keywords (“esophageal cancer” or “esophageal neoplasm” or “neoplasm, esophageal” or “esophagus neoplasm” or “esophagus neoplasms” or “neoplasm, esophagus” or “neoplasms, esophagus” or “neoplasms, esophageal” or “cancer of esophagus” or “cancer of the esophagus” or “esophagus |

| | | | | |
|---|---|---|---|---|
| | | | | cancer” or “cancer, esophagus” or “cancers, esophagus” or “esophagus cancers” or “esophageal cancer” or “cancer, esophageal” or “cancers, esophageal” or “esophageal cancers”) and (“sarcopenia” or “muscular atrophy” or “aging” or “senescence” or “biological aging” or “aging, biological” or “atrophies, muscular” or “atrophy, muscular” or “muscular atrophies” or “atrophy, muscle” or “atrophies, muscle” or “muscle atrophies”). Studies reporting relationship between sarcopenia and esophageal cancer were analyzed. " |
| Search strategy | 7 | Present the full search strategies for all databases, registers and websites, including any filters and limits used | Abstract | Page1, "Methods: A narrative review was performed in PubMed and Web of Science using the keywords (“esophageal cancer” or “esophageal neoplasm” or “neoplasm, esophageal” or “esophagus neoplasm” or “esophagus neoplasms” or “neoplasm, esophagus” or “neoplasms, esophagus” or “neoplasms, esophageal” or “cancer of esophagus” or “cancer of the esophagus” or “esophagus cancer” or “cancer, esophagus” or “cancers, esophagus” or “esophagus cancers” or “esophageal cancer” or “cancer, |

| | | | | |
|---|---|---|---|---|
| | | | | esophageal” or “cancers, esophageal” or “esophageal cancers”) and (“sarcopenia” or “muscular atrophy” or “aging” or “senescence” or “biological aging” or “aging, biological” or “atrophies, muscular” or “atrophy, muscular” or “muscular atrophies” or “atrophy, muscle” or “atrophies, muscle” or “muscle atrophies”). Studies reporting relationship between sarcopenia and esophageal cancer were analyzed. " |
| Selection process | 8 | Specify the methods used to decide whether a study met the inclusion criteria of the review, including how many reviewers screened each record and each report retrieved, whether they worked independently, and if applicable, details of automation tools used in the process. | | not applicable |
| Data collection process | 9 | Specify the methods used to collect data from reports, including how many reviewers collected | | not applicable |

| | | | | |
|---|---|---|---|---|
| | | data from each report, whether they worked independently, any processes for obtaining or confirming data from study investigators, and if applicable, details of automation tools used in the process. | | |
| Data items | 10a | List and define all outcomes for which data were sought. Specify whether all results that were compatible with each outcome domain in each study were sought (e.g. for all measures, time points, analyses), and if not, the methods used to decide which results to collect. | | not applicable |
| | 10b | List and define all other variables for which data were sought (e.g. participant and intervention | | not applicable |

| | | | | |
|---|---|---|---|---|
| | | characteristics, funding sources). Describe any assumptions made about any missing or unclear information. | | |
| Study risk of bias assessment | 11 | Specify the methods used to assess risk of bias in the included studies, including details of the tool(s) used, how many reviewers assessed each study and whether they worked independently, and if applicable, details of automation tools used in the process. | | not applicable |
| Effect measures | 12 | Specify for each outcome the effect measure(s) (e.g. risk ratio, mean difference) used in the synthesis or presentation of results. | | not applicable |
| Synthesis methods | 13a | Describe the processes used to decide which studies were eligible for each synthesis (e.g. tabulating the study intervention | | not applicable |

| | | | | |
|---|---|---|---|---|
| | | characteristics and comparing against the planned groups for each synthesis (item #5)). | | |
| | 13b | Describe any methods required to prepare the data for presentation or synthesis, such as handling of missing summary statistics, or data conversions. | | not applicable |
| | 13c | Describe any methods used to tabulate or visually display results of individual studies and syntheses. | | not applicable |
| | 13d | Describe any methods used to synthesize results and provide a rationale for the choice(s). If meta-analysis was performed, describe the model(s), method(s) to identify the presence and extent of statistical | | not applicable |

| | | | | |
|---|---|---|---|---|
| | | heterogeneity, and software package(s) used. | | |
| | 13e | Describe any methods used to explore possible causes of heterogeneity among study results (e.g. subgroup analysis, meta-regression). | | not applicable |
| | 13f | Describe any sensitivity analyses conducted to assess robustness of the synthesized results. | | not applicable |
| Reporting bias assessment | 14 | Describe any methods used to assess risk of bias due to missing results in a synthesis (arising from reporting biases). | | not applicable |
| Certainty assessment | 15 | Describe any methods used to assess certainty (or confidence) in the body of evidence for an outcome. | | not applicable |
| **Results** | | | | |
| Study selection | 16a | Describe the results of the search and selection process, from the number of records identified in | | not applicable |

| | | | | |
|---|---|---|---|---|
| | | the search to the number of studies included in the review, ideally using a flow diagram. | | |
| | 16b | Cite studies that might appear to meet the inclusion criteria, but which were excluded, and explain why they were excluded. | | not applicable |
| Study characteristics | 17 | Cite each included study and present its characteristics. | | Page28, Table1,2,3 |
| Risk of bias in studies | 18 | Present assessments of risk of bias for each included study. | | not applicable |
| Results of individual studies | 19 | For all outcomes, present, for each study: (a) summary statistics for each group (where appropriate) and (b) an effect estimate and its precision (e.g. confidence/credible interval), ideally using structured tables or plots. | 2. Prevalence of sarcopenia in esophageal cancer | Page5, " Among the included studies (Table 1), the average prevalence of sarcopenia in esophageal cancer was found to be 46.3% ± 19.6%. " |
| | | | 3. The role of sarcopenia in the prognosis of surgical treatment of esophageal cancer | Page 5, "Numerous studies have demonstrated that preoperative sarcopenia not only increases the risk of complications such as pulmonary issues and mortality in older adults, but also leads to extended hospital stays and reduced survival rates. Numerous studies have demonstrated that preoperative sarcopenia not only increases the risk of complications |

| | | | | |
|---|---|---|---|---|
| | | | | such as pulmonary issues and mortality in older adults, but also leads to extended hospital stays and reduced survival rates."<br>Page 6, "Apart from its impact on surgical complications, preoperative sarcopenia has also been linked to long-term prognosis." is a presentation of the results; |
| | | | 4. Chemotherapy and sarcopenia | Page 7," 4.1 Chemotherapy-induced sarcopenia"<br>Page 9," Table 2 lists the studies related to the effect of sarcopenia on chemotherapy in esophageal cancer. Based on the a forementioned studies, it is evident that sarcopenia independently indicates reduced overall survival, disease-free survival, and recurrence-free survival in patients with esophageal cancer who undergo chemotherapy. Furthermore, sarcopenia increases the incidence of toxic reactions, mucositis, fever, and lymphopenia, consequently leading to perioperative complications, an elevated risk of postoperative recurrence rates, and postoperative mortality. " |
| | | | 5. Potential mechanisms of esophageal | Page9, each subheading is a result section<br>Page9, 5.1 Malnutrition |

| | | | | |
|---|---|---|---|---|
| | | | cancer-associated sarcopenia | Page10, 5.2 Lack of exercise lifestyle<br>Page10, 5.3 Inflammation<br>Page10, 5.4 Chemotherapy causes sarcopenia<br>Page11, 5.5 Other signaling pathways |
| | | | 6. Predicting esophageal cancer prognosis with a simple indicator in the diagnosis of sarcopenia | Page 12," Besides assessing the presence of sarcopenia according to diagnostic criteria and gauging its prognostic significance, researchers have been particularly intrigued by the extent of skeletal muscle mass reduction during treatment or post-surgery. In instances where standardized tests fail to meet the criteria for diagnosing sarcopenia, employing alternative, efficient methods like HGS, 5-SCT, GS, 6MWD, and CC to predict prognosis is highly desirable. " |
| | | | 7. Treatment of sarcopenia | 7.1 non-pharmacological treatment<br>7.2 Pharmacologic Treatment |
| | | | 8. Conclusions and future perspectives | the section "8. Conclusions and future perspectives" presents the conclusions and discussion of the entire review. |
| Results of syntheses | 20a | For each synthesis, briefly summarise the characteristics and risk of bias among contributing studies. | | not applicable |
| | 20b | Present results of all | | not applicable |

| | | | | |
|---|---|---|---|---|
| | | statistical syntheses conducted. If meta-analysis was done, present for each the summary estimate and its precision (e.g. confidence/credible interval) and measures of statistical heterogeneity. If comparing groups, describe the direction of the effect. | | |
| | 20c | Present results of all investigations of possible causes of heterogeneity among study results. | | not applicable |
| | 20d | Present results of all sensitivity analyses conducted to assess the robustness of the synthesized results. | | not applicable |
| Reporting biases | 21 | Present assessments of risk of bias due to missing results (arising from reporting biases) for each synthesis assessed. | | not applicable |
| Certainty of evidence | 22 | Present assessments of certainty (or | | not applicable |

| | | | | |
|---|---|---|---|---|
| | | confidence) in the body of evidence for each outcome assessed. | | |
| **Discussion** | | | | |
| Discussion | 23a | Provide a general interpretation of the results in the context of other evidence. | 2. Prevalence of sarcopenia in esophageal cancer | Page5, " However, the prevalence of sarcopenia in patients with esophageal cancer varies considerably due to differences in study populations, age, diagnostic methods, and criteria, and the criteria used to determine the prevalence of sarcopenia varied among the studies in this review, as shown in Table 1, with prevalence rates ranging from 14.4% to 81%. "<br>Page5, " Despite discrepancies in diagnostic criteria and methods, sarcopenia was frequently diagnosed during preoperative examinations in patients with esophageal cancer. Given that esophageal cancer exhibits the highest prevalence of sarcopenia among gastrointestinal tumors, it is imperative to allocate greater attention to this condition in esophageal cancer patients. " |
| | | | 5. Potential mechanisms of esophageal cancer-associated sarcopenia | Page9, this section is discussing the possible causes of sarcopenia. |

| | | | | |
|---|---|---|---|---|
| | | | 6. Predicting esophageal cancer prognosis with a simple indicator in the diagnosis of sarcopenia | Page12, "Besides assessing the presence of sarcopenia according to diagnostic criteria and gauging its prognostic significance, researchers have been particularly intrigued by the extent of skeletal muscle mass reduction during treatment or post-surgery. In instances where standardized tests fail to meet the criteria for diagnosing sarcopenia, employing alternative, efficient methods like HGS, 5-SCT, GS, 6MWD, and CC to predict prognosis is highly desirable. " |
| | 23b | Discuss any limitations of the evidence included in the review. | 2. Prevalence of sarcopenia in esophageal cancer | Page5, " However, the prevalence of sarcopenia in patients with esophageal cancer varies considerably due to differences in study populations, age, diagnostic methods, and criteria, and the criteria used to determine the prevalence of sarcopenia varied among the studies in this review, as shown in Table 1, with prevalence rates ranging from 14.4% to 81%. "<br>Page5, " Despite discrepancies in diagnostic criteria and methods, sarcopenia was frequently diagnosed during preoperative examinations in patients with esophageal cancer. Given that esophageal cancer exhibits the highest prevalence of sarcopenia |

| | | | | |
|---|---|---|---|---|
| | 23d | | | among gastrointestinal tumors, it is imperative to allocate greater attention to this condition in esophageal cancer patients. " |
| | | | 7. Treatment of sarcopenia | Page15, "Despite the existence of studies showcasing the positive effects of the aforementioned drugs in sarcopenia patients, their efficacy remains a subject of controversy. Furthermore, the optimal dosage and potential side effects of these drugs require further investigation through additional studies. The pharmacological treatment of sarcopenia necessitates more extensive exploration and clinical trials to scientifically evaluate the efficacy of these drugs. " |
| | | | 8. Conclusions and future perspectives | Page15, "To date, the majority of studies investigating sarcopenia in esophageal cancer patients have primarily relied on retrospective approaches, severely constraining their ability to comprehensively depict patient populations. Consequently, our understanding of the underlying mechanisms linked to heightened adverse outcomes remains limited" |
| | 23c | Discuss any limitations of the review processes used. | | not applicable |
| | 23d | Discuss implications | 2. Prevalence | Page5, " Despite discrepancies in |

| | | | | |
|---|---|---|---|---|
| | | of the results for practice, policy, and future research. | of sarcopenia in esophageal cancer | diagnostic criteria and methods, sarcopenia was frequently diagnosed during preoperative examinations in patients with esophageal cancer. Given that esophageal cancer exhibits the highest prevalence of sarcopenia among gastrointestinal tumors, it is imperative to allocate greater attention to this condition in esophageal cancer patients. " |
| | | | 3. The role of sarcopenia in the prognosis of surgical treatment of esophageal cancer | Page 6, "Consequently, the routine evaluation and accurate diagnosis of sarcopenia in older adults has been linked to long-term prognosis. diagnosis of sarcopenia in esophageal cancer patients can assist clinicians in tailoring treatment plans, providing timely nutritional support, and ultimately improving short-term and long-term patient outcomes, as well as the overall prognosis of esophageal cancer."<br>Page 7," The prognosis of postoperative muscle loss in esophageal cancer has received limited attention and the timing of postoperative detection of sarcopenia varies considerably across studies, but each of these studies consistently demonstrates a substantial association between postoperative muscle loss or reduced skeletal muscle mass and |

| | | | | |
|---|---|---|---|---|
| | | | | poor prognosis, and more prospective cohort studies are needed to demonstrate this association." |
| | | | 4. Chemotherapy and sarcopenia | Page 9," Early implementation of appropriate nutritional intervention prior to treatment may improve prognosis" |
| | | | 6. Predicting esophageal cancer prognosis with a simple indicator in the diagnosis of sarcopenia | Page12, "Besides assessing the presence of sarcopenia according to diagnostic criteria and gauging its prognostic significance, researchers have been particularly intrigued by the extent of skeletal muscle mass reduction during treatment or post-surgery. In instances where standardized tests fail to meet the criteria for diagnosing sarcopenia, employing alternative, efficient methods like HGS, 5-SCT, GS, 6MWD, and CC to predict prognosis is highly desirable. " |
| | | | 7. Treatment of sarcopenia | Page15, "Despite the existence of studies showcasing the positive effects of the aforementioned drugs in sarcopenia patients, their efficacy remains a subject of controversy. Furthermore, the optimal dosage and potential side effects of these drugs require further investigation through additional studies. The pharmacological treatment of sarcopenia necessitates more |

| | | | | |
|---|---|---|---|---|
| | | | | extensive exploration and clinical trials to scientifically evaluate the efficacy of these drugs. " |
| | | | 8. Conclusions and future perspectives | Page15, "Hence, it is imperative to conduct more prospective evaluations on sarcopenia in individuals afflicted with esophageal cancer. These evaluations will enable us to establish a more profound comprehension of the correlation between sarcopenia, characterized by the depletion of skeletal muscle mass or strength, and adverse outcomes or post-treatment complications. Furthermore, they will facilitate the development of precise and personalized interventions based on the findings, thereby enhancing outcomes in high-risk populations[114]. By performing requisite assessments of sarcopenia in esophageal cancer patients, we can devise optimal treatment strategies that rectify the sarcopenic condition prior to surgery or chemotherapy through nutritional support and exercise, adjuvant therapy, and meticulous postoperative monitoring[115]. This comprehensive approach aims to augment the quality of life for patients with esophageal cancer while simultaneously alleviating |

| | | | | |
|---|---|---|---|---|
| | | | | the healthcare burden on society." |
| **Other information** | | | | |
| Registration and protocol | 24a | Provide registration information for the review, including register name and registration number, or state that the review was not registered. | | not applicable |
| | 24b | Indicate where the review protocol can be accessed, or state that a protocol was not prepared. | | not applicable |
| | 24c | Describe and explain any amendments to information provided at registration or in the protocol. | | not applicable |
| Support | 25 | Describe sources of financial or non-financial support for the review, and the role of the funders or sponsors in the review. | Funding | Page17, "This work was supported by Natural Science Foundation General Program of Hunan Province (2022JJ40830), Natural Science Foundation General Program of Changsha City (kq2014290) and National Multidisciplinary Cooperative Diagnosis and Treatment Capacity Building Project for Major Diseases (Lung Cancer, grant number: z027002). " |
| Competing interests | 26 | Declare any competing interests | Competing interests | Page17,"The authors declare that they have no competing interests." |

| | | | | |
|---|---|---|---|---|
| | | of review authors. | | |
| Availability of data, code and other materials | 27 | Report which of the following are publicly available and where they can be found: template data collection forms; data extracted from included studies; data used for all analyses; analytic code; any other materials used in the review. | | not applicable |
